# Effects of guided counseling during pregnancy on birth weight of newborns in West Gojjam Zone, Ethiopia: a cluster-randomized controlled trial

**DOI:** 10.1186/s12887-020-02363-8

**Published:** 2020-10-06

**Authors:** Yeshalem Mulugeta Demilew, Getu Degu Alene, Tefera Belachew

**Affiliations:** 1 Department of Nutrition, School of Public Health, College of Medicine and Health Sciences, Bahir Dar University, P.O. Box 79, Bahir Dar, Ethiopia; 2 Department of Biostatics and Epidemiology, School of Public Health, College of Medicine and Health Sciences, Bahir Dar University, P.O. Box 79, Bahir Dar, Ethiopia; 3 Department of Nutrition and Dietetics, Faculty of Public Health, Jimma University|, P.O. Box 378, Jimma, Ethiopia

**Keywords:** Birth weight, Guided counseling, Intervention

## Abstract

**Background:**

The high proportion of birth weight in Ethiopia is hypothesized to be due to inadequate maternal diet which is associated with poor nutrition education during pregnancy. There was no study that evaluated the effect of nutrition education on birth weight in the study area. This study aimed to assess the effects (overall, direct and indirect effects) of guided counseling on the birth weight of neonates.

**Methods:**

A two-arm parallel cluster randomized controlled community trial was conducted from May 1, 2018, to April 30, 2019, in West Gojjam Zone, Northwest Ethiopia. At the baseline, 346 pregnant women in the 11 intervention clusters and 348 pregnant women in the 11 control clusters were recruited. However, birth weight was measured from 258 and 272 newborns in the intervention and control groups, respectively.

In the intervention group, counseling was given monthly for four consecutive months in the participant’s homes. Besides, leaflets with key counseling messages were distributed to each woman in the intervention arm. Pregnant women who attended routine nutrition education given by the health system were recruited as control. Dietary practice, nutritional status, and birth weight were the primary, secondary and tertiary outcomes of this intervention. Data were collected using a structured data collection tool. Birth weight was measured within 48 h after birth. Independent sample t-test, linear mixed-effects model, and path analysis were fitted to assess effects of the intervention.

**Results:**

The intra-cluster correlation coefficient was 0.095. The average birth weight of newborns in the intervention group was 0.257 kg higher compared with their counterparts in the control arm (β = 0.257, *P* < 0.001). The direct effect of this intervention on birth weight was 0.17 (β = 0.17, *P*<0.001 ) whereas the indirect effect of this intervention was 0.08 (β = 0.08, *P*<0.001 ).

**Conclusion:**

Counseling using the health belief model and the theory of planned behavior has a positive effect on improving birth weight. The findings suggest the need for enhancing nutrition education of pregnant women through the application of theories to improve birth weight.

**Trial registration:**

Clinical Trials.gov NCT03627156, “Retrospectively registered Jun, 13, 2018”.

## Background

According to the World Health Organization (WHO), low birth weight (LBW) is weight at birth less than 2500 g irrespective of the gestational age of the neonate. Whereas macrosomic babies weigh more than 4000 g at birth [1, 2]. LBW is a key indicator of the progress towards the achievement of the global nutrition targets [3] since a 30% reduction in the number of LBW live births between 2012 and 2025 is one of the six global nutrition targets [4]. However, progress in reducing the prevalence of LBW is unsatisfactory (the average annual rate of reduction is 1.23% per year) to reach the global nutrition targets as it requires an average annual rate of reduction of 2.74% per year [5].

The magnitude of LBW is high throughout the globe affecting 20.5 million neonates in 2015 with 91% from middle and low-income countries [5]. Nearly three of four LBW newborns live in Southern Asia and Sub-Saharan Africa [5].

In Ethiopia, the prevalence of LBW ranged from 7.8% [6] in Jimma to 18% in the Kembata-Tembaro Zone [7]. According to a prospective study done in the southwestern part of Ethiopia, the incidence of LBW was also 17.88 per 100 births [8]. Moreover, the prevalence of LBW was 22.2% in the Amhara region where the study was conducted [1]. Ethiopian demographic and health survey report showed an increasing trend of the prevalence of LBW from 11% in 2011 to 13% in 2016 [1].

LBW is a known underlying cause of neonatal morbidity and mortality. From all neonatal deaths, over 80% occurred in LBW newborns [5]. According to the WHO estimate of 2014, LBW was the underlying cause of 27,243 deaths (4.53% of overall death) in Ethiopia [9].

Moreover, survivors also have a higher probability of being stunted and having impaired cognitive function [10] which in turn can negatively affect school performance, job opportunities, and productivity later in life [11]. LBW also increases the likelihood of developing chronic noncommunicable diseases such as type 2 diabetes mellitus, cardiovascular diseases, hypertension, and cancer [12, 13]. Not only, LBW babies but also macrosomic babies have a high risk of dying during the neonatal period [14], indicating that any extremes of birth weight have an untoward effect on child survival.

Factors associated with low birth weight were having unemployed mother, lack of antenatal care visit, having unintended pregnancy, rural residence, birth interval less than 2 years, previous history of having low birth weight baby, maternal undernutrition, lack of nutrition counseling, not taking an additional meal during pregnancy, pregnancy complication, not taking an iron supplement and preterm delivery [7, 15–17].

Poor maternal diet is one of the several contributing factors to LBW [15–18]. Women who did not take an adequate meal during pregnancy were more likely to have LBW neonate than their counterparts [19–23]. Despite its serious consequences on birth weight and other birth outcomes, nutrient intakes of Ethiopian pregnant women were less than the recommended amounts for several key nutrients [24, 25]. This is hypothesized to be due to poor dietary habits of pregnant women since the majority of pregnant women in the country take a cereal-based monotonous diet low in nutrient content and with poor bioavailability [26–28].

High prevalence of LBW related to inadequate nutrient intakes of pregnant women warrant the importance of implementing preventive strategies to reduce low birth weight. Moreover, previous researchers in Ethiopia suggested enhancing nutrition education during pregnancy [16, 17] as has potential to reduce the incidence of LBW [29, 30].

In Ethiopia, nutrition education is given during antenatal care visits and at the community level [31] by health professionals and health extension workers focusing on the need that pregnant women should take one additional meal from available foods. During counseling, they do not consider the difference in nutrient requirements based on the trimester of pregnancy and pre-pregnancy nutritional status. Moreover, they do not take difference in socioeconomic status and dietary habits of different pregnant women into account. This type of education was ineffective to improve the dietary practices of pregnant women [31]. As a result, the prevalence of low birth weight is very high in the country [1, 26, 28]. Therefore, the effect of nutrition education given by the routine health system should be investigated, and possible remedial measures need to be taken.

In this intervention, education was given based on the trimester of pregnancy, dietary habits, and socioeconomic status of pregnant women. A full description of the intervention can be found in the methods session.

Since multiple theories and models are needed to fully explain dietary behavior [32], in this study counseling was given using the health belief model (HBM) and the theory of planned behavior (TPB). The HBM is a behavioral change model designed to explain and forecast health-related behaviors. Its constructs are perceived susceptibility, severity, benefits, and barriers. When an individual perceives that he/she is susceptible to a specific problem, perceives a problem is serious, and perceives benefits outweigh the barriers, he/she takes measures to change behavior. Having confidence and cue to perform a specific behavior are the other important components of the HBM to bring behavioral change [33].

The TPB suggests that the probability of an individual performing a specific behavior is determined by the strength of his or her intention towards a specific behavior. Attitude, subjective norms, and behavioral control regarding a specific behavior are determinants of intention to bring behavioral change [34]. The objective of this study was to assess the effects (overall, direct and indirect effects) of guided counseling on birth weight of neonates in West Gojjam Zone.

## Methods

### Study design and setting

The intervention was implemented in West Gojjam Zone, Ethiopia, considering the principles of the Helsinki Declaration and the requirements of Good Clinical Practice [35]. The total population of the Zone during data collection was 2,641,240 of which 50.7% were females. A two-arm parallel cluster randomized controlled community trial was conducted from May 1, 2018, to April 30, 2019, to assess the effects of guided counseling on the birth weight of the newborns. A detailed explanation of the study area has been described previously [31].

Pregnant women within the first 4 months of pregnancy who had planned to give birth in the study area were included in this study. As hypertensive’s and cases of diabetes mellitus have different meal plans from normal cases, they were excluded from this study. Full details regarding the study participants and their enrollment are described elsewhere [31].

**Fig. 1:**
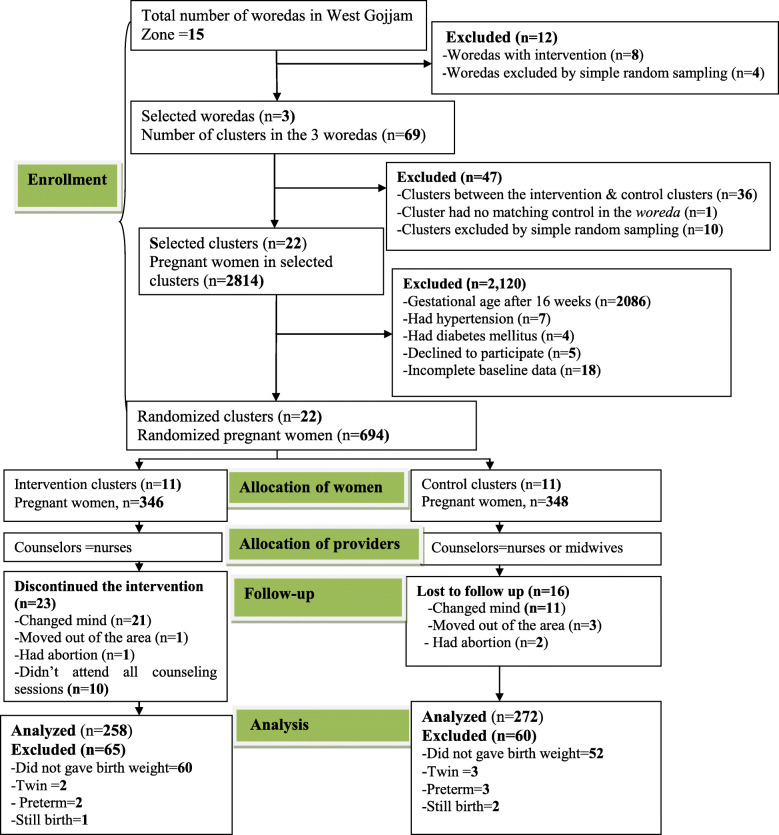
CONSORT flow chart showing the allocation of the study participants through the trial according to the criteria recommended in the CONSORT extension guideline

G power 3.1.9.2 software was used to estimate the sample size [36, 37]. The following assumptions were used to calculate the required sample size: assuming a precision of 5%, a power of 85%, an effect size of 0.5, and mean birth weight change of 100 g over the study period in the intervention group (from 3090 g- 3190 g) [38, 39]. A design effect of 2 was used, and a 10% loss to follow up was taken into account. The calculated sample sizes were 128 and 132 women in the intervention and control groups, respectively. However, sample size determined using G power for the primary outcome of this trial (dietary practice) gave the largest sample sizes (346 women in the intervention group and 348 women in the control group), which were included in the trial. Due to problems reported in Fig. 1, the actual data were collected from 249 women in the intervention group and 272 women in the control arm.

The approval letter was obtained from the Ethical Review Committee of Bahir Dar University (IRB#092/18–04) to conduct the trial. The literate woman signed informed consent, and women who couldn’t sign gave fingerprints prior to conducting the study. The trial was registered in the Clinical Trials.gov NCT03627156 “Retrospectively registered”. The CONSORT extension flow chart and CONSORT checklist of the trial were prepared based on the Consolidated Standards of Reporting Trials (CONSORT) statement (Fig. 1 and Additional file 1) [40, 41].

### Recruitment and randomization

West Gojjam Zone has fifteen Woredas, of which eight had nutrition education intervention on infant feeding practices. These eight Woredas were excluded from this study since women in these Woredas may not be representative of women in the nonintervention area. Three *Woredas* were selected using a simple random sampling (SRS) method from the seven eligible *Woredas*. First, a list of noncontiguous Kebeles was prepared using a map in different colors in consultation with a mapping office. Non-selected kebeles (buffer zone) were found between the intervention and control Kebeles to prevent the probability of message communication [39].

Then the Kebeles (clusters) were randomly allocated to intervention and control arms using the lottery method in a 1:1 ratio. All eligible pregnant women within the first 4 months of gestation in the selected Kebeles were included in the study after screening through a home-to-home visit.

### Intervention and fidelity check

Full details of the intervention and fidelity check are found elsewhere [42]. Community-based counseling about maternal diet was implemented in this study. Counseling was guided by a counseling protocol, the HBM, and the TPB. The counseling guide was prepared by reviewing the recommendations of WHO and the Ministry of Health of Ethiopia [43, 44]. Counseling protocol was attached as Additional file 2.

The core contents of the counseling protocol were increasing the amount and frequency of meals with gestational age, and diversity of meals. Iron/folic acid supplement and iodized salt utilization were also included in the counseling protocol. Moreover, reducing workload, taking day time rest, infection prevention methods, and health care services utilization were the contents of the counseling protocol.

The result of taking insufficient nutrients, susceptibility to, and severity of the effects of inadequate nutrient intakes were also discussed during counseling. The benefits and barriers of eating sufficient amount and balanced diet were included in the counseling protocol. Attitude, subjective norms, self-efficacy, perceived control, intention, knowledge, and dietary practice were assessed during each counseling session, and counseling was given accordingly.

Individual nutrition counseling was given monthly at women’s homes on religious holidays and weekends, using the counseling protocol. Pregnant women in the intervention arm were counseled four times; each counseling session took 40 to 60 min. During counseling, women had an active role in discussing and choosing from locally available, affordable, and acceptable foodstuffs.

The first counseling was implemented within the first 4 months of gestation. It included the importance of taking a balanced diet, selection, preparation, amount, diversity, and frequency of meals. The next two consecutive counseling sessions were delivered during the second trimester of pregnancy and covered the full contents of the counseling protocol. The fourth counseling was delivered during the early third trimester of pregnancy to fill the identified gaps during counseling.

Counseled women were provided leaflets with the core messages of the trial in Amharic (local) language and appropriate pictures. For women who couldn’t read, someone at home or in the neighborhood who could read was recommended to read the leaflet to the woman and other family members. Women in the control arm received nutrition education given by the health care system. The routine practice by the health care system is advising pregnant women to eat one additional meal from foods available at home compared to their meals before pregnancy. The education was given during ANC visit and at the community through a home visit and women’s conference. Nutrition counseling was given in the health system guide on key nutrition practices during pregnancy which was attached as Additional file 3.

Counselors and supervisors of the counseling process were BSc nurses and MSc nutritionists, respectively. The counseling team was trained for 3 days using a training manual. Furthermore, they were given a one-day supplementary training after 60 days of the trial to keep up their proficiency in counseling.

Fidelity of the intervention was evaluated using criteria for best practice recommendations developed by the National Institutes of Health Behavioral Change Consortium [45, 46]. The intervention design, training of counselors, counseling process, receipt of intervention, and enactment of skills gained from the intervention were assessed using the criteria [46].

The intervention was guided by theory and counseling guide. The counseling process was pretested before the implementation. The frequencies and the duration of counseling were approximately similar for all women to make the counseling process standardized. Counselors were trained together and pre- and post-training tests were given. One process evaluator assessed the counseling process using a ‘yes/no’ rating system on items related to counseling.

Maternal knowledge on diet during pregnancy was also assessed through enquiring about their understanding of intervention messages. Moreover, women’s skill was determined by observing a demonstration of preparing and taking diversified meals. Women’s practices of increasing frequency of meal, portion size, iodized salt and iron/folic acid use were also assessed using a checklist.

Counselors and their supervisors met monthly to discuss and propose possible solutions to the challenges encountered during counseling. Participants, counselors, and data collectors were not aware of the study hypotheses. Furthermore, the data entry clerk was blinded by coding the groups until analysis. The supervisors and the investigators closely supervised the counseling process.

### Data collection and measurement

Structured questionnaires on socio-demographic variables, obstetric characteristics, and dietary practices were prepared based on literature [1, 47]. Data on the socio-demographic and obstetric variables were collected at the baseline. Data on dietary practice, mid-upper arm circumference, and women’s weight were collected before and after the trial. Six nurses collected data through face-to-face interviews at the participants’ homes.

Data collectors and supervisors were employed and trained for 3 days. Supervisors and the principal investigator followed the data collection process closely. The data collection team met daily to discuss and solve difficulties. Dietary practice and nutritional status of pregnant women were primary and secondary outcomes whereas birth weight was the third outcome of this intervention.

Birth weight was measured within 48 h of life using balanced digital Seca scales, read to the nearest 100 g [48]. The scales were calibrated before each measurement using an object of known weight. Moreover, the reading on each scale was adjusted to zero levels before weighing each newborn. During weighing the women wore light clothes. Gestational weight gain was calculated by subtracting baseline weight from the end-line weight.

To assess the Mid Upper Arm Circumference (MUAC) of the respondents, the distance from the acromion to olecranon processes was measured while the respondents’ elbows were flexed to 90^0^. The midpoint was marked, and measuring tape was placed snugly around the arm at the midpoint mark while hanging arm freely.

Dietary data were collected using the food frequency questionnaire (FFQ) that contained 54 food items. Food items of the FFQ were categorized into nine food groups (1. cereals, roots, and tubers; 2. vitamin-A-rich fruits and vegetables; 3. other fruits; 4. other vegetables; 5. legumes and nuts; 6. meat, poultry, and fish; 7. fats and oils; 8.dairy; and 9. eggs) [31]. The consumer of a food group was defined as a woman who eats one of the food items for the food group within 1 week. Then for each food group, a consumer was coded as “1” and nonconsumer coded as “0”. Finally, the food groups were summed to generate a dietary diversity score (DDS), which was rank ordered into tertiles [31].

Count of each food items the women ate within a week was used to compute the mean food variety score (FVS). Respondents who consumed above the mean food variety were labeled as having good FVS, or else having poor FVS [49]. Count of the frequency of each animal source food (ASF) the respondents ate within the days of a week was classified into three parts. Women who took the highest part were considered as taking high frequency of ASF consumption, otherwise having a low frequency of ASF consumption [49].

The sums of FVS, ASF consumption, DDS, and frequency of meals were used to assess the dietary practice of pregnant women. The Principal Component Analysis (PCA) was used to generate the wealth index. The availability of a latrine, source of water, household resources, livestock, and farmland ownership were variables included in the PCA. The responses of all non-dummy variables were categorized into three parts. The highest score was labeled as one and the two lower values were coded as zero. Factor scores were produced using variables having a commonality value of above 0.5. The wealth indexes of households were produced using the scores of the first principal component. The wealth index was categorized into five as the poorest, poor, medium, rich, and richest [31].

The sums of composite questions were used to assess each HBM and TPB constructs such as perceived susceptibility, perceived severity, perceived benefits, perceived barriers, attitude, subjective norms, intention, and knowledge on maternal diet.

Level one variables were intervention, crop production, and source of drinking water whereas MUAC at the baseline, maternal age, educational status, source of drinking water, sex of the child, pre-intervention dietary practice, wealth index, and decision making on monetary resources were level one variables.

### Data management and analysis

Multiple births, preterm deliveries, and stillbirths were excluded from the analysis. A chi-square test was used to compare the baseline characteristics of the two groups. The birth weight difference between the two groups was compared using independent samples t-tests.

Since the cluster sampling technique was used, birth weights were nested within the clusters. Taking this into consideration the linear mixed-effects model was used to analyze the effect of the intervention on birth weight. First, the empty model was fitted to determine the need for assessing random effects at the cluster level. Next, the full model which included both level one and level two variables in addition to cluster-specific random effects were fitted. Due to the fact that similar counselor counseled women in the same cluster; the intervention was included as a level two variable. This study was conducted among women in the rural area where the source of drinking water and crop production is similar to women in the same cluster. Due to this crop production and source of drinking water were also considered as a level two variables. The model was adjusted for level one variables such as MUAC at baseline, maternal age, educational status, sex of the child, pre-intervention dietary practice, wealth index, and decision making on monetary resources.

Moreover, the intervention was given about dietary practice, iron/folic acid supplement use, infection prevention measures, and health service utilization. This type of counseling has both direct and indirect effects on birth weight. Taking this into consideration, path analysis was also performed to evaluate the direct and indirect effects of guided counseling on birth weight [50]. Birth weight was the outcome variable and intervention was the predictor of the outcome. Dietary practice and gestational weight gain were mediators of the intervention effect. All other level one and level two variables listed above were also adjusted during path analysis.

This regression-based path analysis was implemented using the PROCESS macro version 3.4 for SPSS, developed by Andrew F Hayes [50]. The pre-requisites, namely linearity, independence, normality, and homoscedasticity were checked before running the model. Shapiro Wilk’s test (*p >* 0.05) showed that birth weight was approximately normally distributed. Durbin-Watson *<* 4 and scatter plots also showed that observations are independent and linear, respectively. The variance inflation factor (VIF) was less than five. Before path analysis, a linear regression model was fitted to examine the association between predictors and birth weight.

Then, the mediation effects of dietary practice and gestational weight gain were assessed in serial multiple mediation analysis. This model also adjusted for the abovementioned predictors. *P*-value *<* 0.05 and 95% CI were used to assess statistical significance. All statistical analyses were performed using SPSS package version 23.

## Results

### Socio-demographic and obstetric characteristics of pregnant women

Birth weight of 530(intervention group =258, control group =272) neonates was measured within 48 h. The two groups have comparable baseline socio-demographic and obstetric characteristics (Table 1).

**Table 1:** Baseline socio-demographic and obstetric characteristics of pregnant women in West Gojjam Zone, Ethiopia

Variables	Intervention group (n_1_ = 258)	Control group (n_2_ = 272)	***P***
	Frequency (%)	Frequency (%)	
**Number of clusters**	11	11	
**Age (years)**			
< 20	21(8.2)	13(4.8)	0.138
20–24	47(18.2)	58(21.3)	
25–29	82(31.8)	68(25.0)	
30–34	62(24.0)	71(26.1)	
> =35	46(17.8)	62(22.8)	
**Religion**			
Orthodox	256(99.2)	270(99.3)	0.958
Muslim	2(0.8)	2(0.7)	
**Educational status**			
Couldn’t read and write	199(77.1)	195(71.7)	0.060
Can read and write	17(6.6)	15(5.5)	
Primary education	34(13.2)	39(14.3)	
Secondary education	8(3.1)	23(8.5)	
**Occupational status**			
Housewife	120(46.5)	143(52.6)	0.163
Farmer	138(53.5)	129(47.4)	
**Marital status**			
Married	253(98.1)	271 (99.6)	0.197
Unmarried/ Divorced	5 (1.9)	1(0.4)	
**Husband education (*n*** **= 253,** ***n*** **= 271)**			
No formal education	190(75.1)	202(74.5)	0.988
Primary education	45(17.8)	49(18.1)	
Secondary and above education	18(7.1)	20(7.4)	
**Wealth index**			
Poorest	41(15.9)	51(18.7)	
Poor	56(21.7)	53(19.5)	0.767
Medium	58(22.5)	54(19.9)	
Rich	52(20.2)	62(22.8)	
Richest	51(19.7)	52(19.1)	
**Family Size**			
< 5	137 (53.1)	133 (48.9)	0.333
> =5	121 (46.9)	139 (51.1)	
**Number of pregnancy**			
1	57 (22.1)	57 (21.0)	0.602
2–3	75 (29.1)	68(25.0)	
4–5	81 (31.4)	90 (33.1)	
> =6	45(17.4)	57 (20.9)	
**Number of delivery**			
0	60 (23.3)	62(22.8)	
1–3	125 (48.4)	118 (43.4)	0.552
4–5	55 (21.3)	68(25.0)	
> =6	18(7.0)	24 (8.8)	
**Sex of the child**			
Male	132 (51.2)	137 (50.4)	0.813
Female	126 (48.8)	135 (49.6)	

*IG* Intervention group, *CG* Control group

### Birth weight and nutritional status of pregnant women

At the endline survey, the mean birth weight of babies born in the intervention group was 3.18 kg (±0.44), while it was 2.92 kg (±0.42) for the control arm showing higher birth weight in the intervention group by 0.26 kg. Mean gestational weight gain was 8.12 kg in the intervention group and 7.17 kg in the control arm (Table 2).

**Table 2:** Comparison of gestational weight gain and birth weight of pregnant women in West Gojjam Zone, Ethiopia using independent sample t-test (intention to treat analysis)

Variables	Intervention	Control	Difference	***P***
	Mean (±SD) (Kg)	Mean (±SD) (Kg)	Mean (SE) (Kg) (95% CI)	
Birth weight	3.18(±0.44)	2.92(±0.42)	0.26(0.03), (0.18,0.33)	< 0.001
Gestational weight gain	8.12(±2.49)	7.17(±2.56)	0.95(0.22), (0.52,1.38)	< 0.001
Gestational age (week)	39.20(±1.26)	38.99(±1.16)	0.20(0.10), (0.001,0.41)	0.049

A higher proportion of the newborns in the control group had low birth weight than the intervention arm (14.7% Vs 6.4%, *p* = 0.002). There were 1.2% of macrosomic babies in the intervention group, whereas, there was no macrosomic baby in the control group. There was a significant difference in the mean gestational age at birth (mean ± SD = 39.20 ± 1.26 Vs 38.99 ± 1.16, *P* = 0.049).

MUAC of the study participants was comparable before the implementation of the intervention (*P* = 0.67). At the end of the trial, counseled women had higher MUAC than their counterparts. More women in the intervention group gave birth in the health institution than women in the control group (*P* = 0.001) (Table 3).

**Table 3:** Comparison of the birth weight of neonates and nutritional status of pregnant women in West Gojjam Zone, Ethiopia

Variables	Intervention (n_**1**_ = 258)	Control (n_**2**_ = 272)	***P***
**Birth weight**			
Low birth weight (< 2500 g)	16 (6.2)	40 (14.7)	0.001
Normal birth weight (2500 g -4000 g)	239 (92.6)	232 (85.3)	
Macrosomic (> 4000 g)	3 (1.2)		
**MUAC before intervention**			
**< 23 cm**	108 (41.9)	109 (40.1)	0.676
**> =23 cm**	150 (58.1)	163 (59.9)	
**MUAC after intervention**			
**< 23 cm**	77 (29.8)	134 (49.3)	< 0.001
**> =23 cm**	181 (70.2)	138 (50.7)	
**Place of delivery**			
Institution	214 (82.9)	193 (71.0)	
Home	44 (17.1)	79 (29.0)	0.001

### Effects of guided counseling on birth weight

The first model (empty model) showed significant variability of the average birth weight across clusters (*p* = 0.02). The intra-cluster correlation coefficient was 0.095. According to the Linear mixed-effects model, the average birth weight in the intervention group increased by 0.257 kg compared with birth weight in the control group (β = 0.257, *P* <0.001)  (Table 4).

**Table 4:** Linear mixed model predicting birth weight among newborns delivered in West Gojjam Zone

Fixed effect						
	Model 1		Model 2		Model 3	
Variables	Estimate (SE)	95% CI	Estimate (SE)	95% CI	Estimate (SE)	95% CI
Intercept	3.05 (0.035)	(2.98,3.12)	2.92 (0.027)	(2.87,2.98)	1.56 (0.239)	(1.09,2.04)
Intervention			**0.261 (0.039)**	(0.17,0.34)	**0.257 (0.041)**	(0.17,0.34)
Crop production						
Cereals					1.00	1.00
Cereals & legume					0.001 (0.056)	(−0.111,0.114)
Cereals, legume, vegetables & fruits					0.024 (0.049)	(−0.073,0.122)
MUAC baseline					0.053 (0.009)	(0.03,0.07)
Dietary practice baseline					0.180 (0.047)	(0.08,0.27)
Being female					0.021 (0.036)	(−0.04,0.09)
Increasing wealth					0.0001 (0.013)	(−0.02,0.02)
Having decision making power					0.005 (0.039)	(−0.07,0.08)
Piped water supply					0.062 (0.039)	(−0.01,0.15)
Having formal education					0.045 (0.050)	(−0.05,0.14)
Age					0.003 (0.003)	(−0.01,0.01)
**Random effect**						
Level two variance	0.0198 (0.008)		0.0009 (0.002)		0.0006 (0.001)	
ICC	0.0954		0.0047		0.0039	
AIC	651.034		629.991		594.473	
Number of parameters	3		5		16	

*SE* Standard error, *CI* Confidence interval, *AIC* Akaike information criterion, *ICC* Intra cluster correlation coefficient

Path analysis also showed a similar result such that after adjusting for the potential confounders**,** the average birth weight in the intervention group increased by 0.26 kg compared with birth weight in the control group (β = 0.26, *P*<0.001 ). The direct effect of this intervention on birth weight was 0.17 (β = 0.17, *P*<0.001) . Whereas, the indirect effect of this intervention was 0.08 (β = 0.088, *P*<0.001 ) (Table 5).

**Table 5:** Mediation analysis of direct and indirect effects of the intervention on birth weight of pregnant women in West Gojjam Zone, Ethiopia

Variables	Estimate (SE)	95% CI
Total effect of the intervention on birth weight	0.260 (0.038)	(0.186,0.335)
Direct effect of the intervention on birth weight	0.172 (0.039)	(0.094,0.249)
Total indirect effect of the intervention on birth weight	0.088 (0.023)	(0.041,0.138)
Indirect effect1	0.046 (0.018)	(0.011,0.084)
Indirect effect2	0.023 (0.012)	(0.001,0.050)
Indirect effect3	0.018 (0.006)	(0.007,0.032)
Effect of the dietary practice on birth weight	0.007 (0.002)	(0.002,0.012)
Effect of gestational weight gain on birth weight	0.049 (0.007)	(0.035,0.063)
Effect of the intervention on dietary practice	6.330 (0.617)	(5.116,7.541)
Effect of the dietary practice on gestational weight gain	0.060 (0.015)	(0.029,0.091)
Effect of the intervention on gestational weight gain	0.483 (0.242)	(0.006,0.959)
MUAC baseline	0.050 (0.008)	(0.032,0.067)
Dietary practice baseline	0.003 (0.002)	(−0.001,0.008)
Being female	0.014 (0.035)	(−0.054,0.083)
Increasing wealth	0.004 (0.013)	(−0.022,0.030)
Having decision making power on monetary use	0.004 (0.038)	(−0.070,0.080)
Piped water supply	0.036 (0.038)	(−0.038,0.0112)
Having formal education	0.025 (0.049)	(−0.072,0.122)
Age	0.003 (0.003)	(−0.003,0.009)
Crop production	0.002 (0.021)	(−0.040,0.045)

Indirect effect key:

Indirect effect 1: shows intervention effect on birth weight through dietary practice

Indirect effect 2: reveals intervention effect on birth weight through gestational weight gain

Indirect effect 3: indicates intervention effect on birth weight through dietary practice and gestational weight gain

## Discussion

The findings of this study confirmed the effectiveness of guided counseling using the health belief model and the theory of planned behavior in improving gestational weight gain and birth weight. Women who got counseling on the maternal diet had better gestational weight gain than women who didn’t get counseling.

A similar finding was reported by the previous study in Bangladesh [51]. This positive effect of the intervention in improving gestational weight gain might be due to the effect of counseling in improving women’s dietary practice [52]. Counseled women were more likely to have appropriate dietary practice than their counterparts. Taking an adequate amount and a good quality diet is a direct determinant of gestational weight gain.

Neonates in the intervention group were heavier than newborns in the control group. The mean birth weight of newborns in the intervention group was 0.26 kg greater than the mean birth weight of neonates in the control group. This result is similar to the study finding in Kenya [30].

After the model was adjusted for potential confounders, the linear mixed-effects model and process model results also revealed higher birth weight in the intervention group compared with the control group. This result is consistent with previous studies reported by different scholars [51, 53].

This difference might be due to the difference between counseling given for the intervention and control groups. In this study, counseling was given based on the health belief model and the theory of planned behavior. Counselors used a counseling guide with the core messages of the intervention. Counseling gave emphasis to increasing portion size, frequency, and diversity of meals with increasing gestational age. Moreover, in the intervention group counselors follow a client-centered approach. They assess women’s, knowledge on diet during pregnancy, availability, and accessibility of foodstuffs and counseled them based on the need of individual pregnant woman. All these have significant contributions in improving the dietary practices of pregnant women. Taking an adequate amount of good quality diet plays a crucial role in improving the birth weight of neonates.

Whereas, health professionals and health extension workers counsel women to take foods available at home by adding one extra meal compared with women’s usual diet. One extra meal may not be enough for all pregnant women during the third trimester. Foods available at home may not be diversified to meet the nutrient demand of all pregnant women. This type of general non-tailored education may not bring positive results.

The direct effect of the intervention on birth weight was 0.18. The possible explanation for the direct positive effect of this intervention could be the core contents of counseling. In addition to the maternal diet, women in the intervention group got counseling about disease prevention and health service utilization. This increased women’s knowledge and disease prevention practices [54, 55]. These healthy practices, in turn, improved birth weight [30].

The intervention effect on birth weight was mediated by women’s dietary practice and gestational weight gain. The indirect effect through path1 (intervention effect through dietary practice) was significant. Previous studies also reported the positive effect of nutrition counseling in improving the dietary practice of pregnant women [52, 56] which, in turn, improved birth weight [57].

Another indirect effect of the intervention was through gestational weight gain (path2). A systematic review of studies about the effect of nutrition interventions on birth weight also reported the indirect effect of nutrition intervention on birth weight [29]. According to a study done in Bangladesh, nutrition intervention improved dietary intake, gestational weight gain, and birth weight of the newborns [58]. Similarly in this study, indirect effect through path3 (intervention effect through dietary practice and gestational weight gain) was also significant.

The possible explanation could be that women in the intervention group got counseling about maternal diet and the importance of gestational weight gain using the HBM and the TPB. This type of counseling increased women’s knowledge about the consequences of poor diet, the benefits of taking a balanced diet, food preparation, and weight gain [54, 55]. This, in turn, improve gestational weight gain and birth weight [30].

The prevalence of low birth weight in the control group was higher than in the intervention group. The 2016 Ethiopian demographic and health survey also reported a 13% prevalence of low birth weight [1]. Studies in Kenya [30] and Bangladesh [51] also reported a lower prevalence of low birth weight in the intervention group than the control arms. The positive effect of the intervention in improving dietary practice and health-seeking behavior might explain this difference.

The results have practical implications in preventing low birth weight. The fact that significantly lower numbers of newborns have LBW in the intervention group implies the need for enacting the community level education through health extension workers using theory enhanced approaches.

Although this study was a large and well-organized randomized controlled trial with good data management, increasing the reliability of the data, it is not free from limitations. The findings couldn’t be generalized to urban dwellers as this study was done among rural residents. The high rate of loss to follow up is also a limitation of this study. Though a loss to follow up is high, the baseline characteristics of the study participants were similar between the two groups.

## Conclusion

Counseling using the health belief model and the theory of planned behavior has a positive effect on improving birth weight. Dietary practice and gestational weight gain were mediators of intervention effect on birth weight. The findings suggest the need for enhancing the nutrition education of pregnant women through the application of the health belief model and the theory of planned behavior to improve birth weight. This study suggests future research to determine the effect of guided counseling starting before conception.

## Supplementary information

**Additional file 1.**

**Additional file 2.**

**Additional file 3.**

## Data Availability

The datasets analyzed during the current study are available from the corresponding author on reasonable request.

## Abbreviations

ANC: Antenatal Care
ASF: Animal Source Food
CONSORT: Consolidated Standards of Reporting Trials
CG: Control Group
DDS: Dietary Diversity Score
FFQ: Food Frequency Questionnaire
FVS: Food Variety Score
HBM: Health Belief Model
IG: Intervention Group
LBW: Low Birth Weight
MUAC: Mid-upper Arm Circumference
PCA: Principal Component Analysis
SD: Standard Deviation
TPB: Theory of Planned Behavior
WHO: World Health Organization

## References

[1] Central Statistical Agency (CSA) [Ethiopia] and ICF (2016). Ethiopia demographic and health survey 2016.

[2] World Health Organization (1992). International statistical classification of diseases and related health problems.

[3] World Health Organization. Global nutrition monitoring framework: operational guidance for tracking progress in meeting targets for 2025; 2017. https://www.who.int/publications/i/item/9789241513609.

[4] World Health Organization (2014). Comprehensive implementation plan on maternal, infant and young child nutrition.

[5] Blencowe H, Krasevec J, Onis M, Black RE, An X, Stevens GA (2019). National, regional, and worldwide estimates of low birthweight in 2015, with trends from 2000: a systematic analysis. Lancet Glob Health.

[6] Seid SS, Tolosa T, Adugna D (2019). Prevalence of low birth weight and associated factor among neonate born at Jimma medical center (JMC), Jimma, South Western Ethiopia. Transl Biomed.

[7] Alemu A, Abageda M, Biruk Assefa GM (2019). Low birth weight: prevalence and associated factors among newborns at hospitals in Kambata-Tembaro zone, southern Ethiopia 2018. Pan Afr Med J.

[8] Wado YD, Afework MF, Hindin MJ (2014). Effects of maternal pregnancy intention, depressive symptoms and social support on risk of low birth weight: a prospective study from southwestern Ethiopia. PLoS One.

[9] World Health Organization (2014). World health ranking: Ethiopia low birth weight.

[10] Gu H, Wang L, Liu L, Luo X, Wang J, Hou F (2017). A gradient relationship between low birth weight and IQ: a metaanalysis. Sci Rep.

[11] Islam MM (2015). The effects of low birth weight on school performance and behavioral outcomes of elementary school children in Oman. Oman Med J.

[12] Nyirenda MJ, Byass P (2019). Pregnancy, programming, and predisposition. Lancet.

[13] Alexander BT, Dasinger JH, Intapad S (2014). Low birth weight: impact on women’s health. Clin Ther.

[14] Kibria GMA, Burrowes V, Choudhury A, Sharmeen A, Ghosh S, Mahmud A (2018). Determinants of early neonatal mortality in Afghanistan: an analysis of the demographic and health survey 2015. Glob Health.

[15] Talie A, Taddele M, Alemayehu M (2019). Magnitude of low birth weight and associated factors among newborns delivered in Dangla primary hospital, Amhara regional state, Northwest Ethiopia, 2017. Hindawi J Pregnancy.

[16] Bililign N, Legesse M, Akibu M (2018). A review of low birth weight in Ethiopia: socio-demographic and obstetric risk factors. Glob J Res Rev.

[17] Siyoum M, Melese T (2019). Factors associated with low birth weight among babies born at Hawassa University comprehensive specialized hospital, Hawassa, Ethiopia. Ital J Pediatr.

[18] Woldeamanuel GG, Geta TG, Mohammed TP, Shuba MB, Bafa TA (2019). Effect of nutritional status of pregnant women on birth weight of newborns at Butajira referral hospital, Butajira, Ethiopia. SAGE Open Med.

[19] Englund-Ögge L, Brantsæter AL, Juodakis J, Haugen M, Meltzer HM, Jacobsson B (2019). Associations between maternal dietary patterns and infant birth weight, small and large for gestational age in the Norwegian mother and child cohort study. Eur J Clin Nutr.

[20] Devaki G, Shobha R (2018). Maternal anthropometry and low birth weight: a review. Biomed Pharmacol J.

[21] Girma S, Fikadu T, Agdew E, Haftu D, Gedamu G, Dewana Z (2019). Factors associated with low birthweight among newborns delivered at public health facilities of Nekemte town, West Ethiopia: a case control study. BMC Pregnancy Childbirth.

[22] Bansal P, Garg S, Upadhyay HP (2019). Prevalence of low birth weight babies and its association with socio-cultural and maternal risk factors among the institutional deliveries in Bharatpur, Nepal. Asian J Med Sci.

[23] Adegboye MB, Zakari S, Ahmed BA, Olufemi GH, Adegboye MB, Zakari S (2018). Knowledge, awareness and practice of infection control by health care workers in the intensive care units of a tertiary hospital in Nigeria. Afr Health Sci.

[24] Asayehu TT, Lachat C, Henauw SD, Gebreyesus SH (2017). Dietary behaviour, food and nutrient intake of women do not change during pregnancy in southern Ethiopia. Matern Child Nutr.

[25] Diddana TZ (2019). Factors associated with dietary practice and nutritional status of pregnant women in Dessie town, northeastern Ethiopia: a community-based cross-sectional study. BMC Pregnancy Childbirth Vol.

[26] Nana A, Zema T (2018). Dietary practices and associated factors during pregnancy in northwestern Ethiopia. BMC Pregnancy Childbirth.

[27] Zerfu TA, Biadgilign S (2018). Pregnant mothers have limited knowledge and poor dietary diversity practices, but favorable attitude towards nutritional recommendations in rural Ethiopia: evidence from community-based study. BMC Nutr.

[28] Aliwo S, Fentie M, Awoke T, Gizaw Z (2019). Dietary diversity practice and associated factors among pregnant women in north East Ethiopia. BMC Res Notes.

[29] KDS L, Ota E, Shakya P, Dagvadorj A, Balogun OO, Peña-Rosas JP (2017). Effects of nutrition interventions during pregnancy on low birth weight: an overview of systematic reviews. BMJ Glob Health.

[30] Nyamasege CK, Kimani-Murage EW, Wanjohi M, Kaindi DWM (2019). Determinants of low birth weight in the context of maternal nutrition education in urban informal settlements, Kenya. J Dev Orig Health Dis.

[31] Demilew YM, Alem GD, Belachew T (2020). Dietary practices and associated factors among pregnant women in west Gojjam zone, Northwest Ethiopia. BMC Pregnancy Childbirth.

[32] Spahn JM, Reeves RS, Keim KS, Laquatra I, Kellogg M, Jortberg B (2010). State of the evidence regarding behavior change theories and strategies in nutrition counseling to facilitate health and food behavior change. J Am Diet Assoc.

[33] Rosenstock IM (1974). The health belief model and preventive health behavior. Health Educ Monogr.

[34] Ajzen I (1985). From intentions to actions: a theory of planned behavior.

[35] World Health Organization: World Medical Association Declaration of Helsinki (2001). Ethical principles for medical research involving human subjects. Bull World Health Organ.

[36] Faul F, Erdfelder E, Lang A-g, Buchner A (2007). G*power 3:a flexible statistical power analysis program for the social, behavioral, and biomedical sciences. Behav Res Methods.

[37] Rolfes SR, Pinna K, Whitney EN (2020). Understanding normal and clinical nutrition: Cengage learning.

[38] Gebremedhin M, Ambaw F, Admassu E, Berhane H (2015). Maternal associated factors of low birth weight: a hospital based cross-sectional mixed study in Tigray, northern Ethiopia. BMC Pregnancy Childbirth.

[39] Chowdhury M, Raynes-Greenow C, Alam A, Dibley MJ (2017). Making a balanced plate for pregnant women to improve birthweight of infants: a study protocol for a cluster randomised controlled trial in rural Bangladesh. BMJ Open.

[40] Schulz KF, Altman DG, Moher D (2010). CONSORORT 2010 statement: updated guidelines for reporting parallel group randomised trials. BMJ Glob Health.

[41] Boutron I, Moher D, Altman DG, Schulz KF, Ravaud P, for the CONSORT Group (2008). Extending the CONSORT statement to randomized trials of nonpharmacologic treatment: explanation and elaboration. Ann Intern Med.

[42] Demilew YM, Alene GD, Belachew T (2020). Effect of guided counseling in nutritional status of pregnant women in west Gojjam zone, Ethiopia: a cluster-randomized controlled trial. Nutr J.

[43] UNICEF. The community infant and young child feeding counseling package: key messages booklet; 2012. https://www.unicef.org/nutrition/files/Key_Message_Booklet_2012_small.pdf.

[44] Federal Democratic Republic of Ethiopia (2016). National Guideline on adolescent, maternal infant and young child nutrition.

[45] Bellg AJ, Borrelli B, Resnick B, Hecht J, Minicucci DS, Ory M (2004). Enhancing treatment fidelity in health behavior change studies: best practices and recommendations from the NIH behavior change consortium. Health Psychol.

[46] Borrelli B (2011). The assessment, monitoring, and enhancement of treatment Fidelity in public health clinical trials. J Public Health Dent.

[47] Ajzen I (1991). The theory of planned behavior. Org Behav Human Decis Process.

[48] Cutland CL, Lackritz EM, Mallett-Moore T, Bardají A, Chandrasekaran R, Lahariya C (2017). Low birth weight: case definition & guidelines for data collection, analysis, and presentation of maternal immunization safety data. Vaccine.

[49] Belachew T, Lindstrom D, Gebremariam A, Hogan D, Lachat C, Huybregts L (2013). Food insecurity, food based coping strategies and suboptimal dietary practices of adolescents in Jimma zone Southwest Ethiopia. PLoS One.

[50] Hayes AF (2018). Introduction to mediation, moderation, and conditional process analysis: a regression-based approach.

[51] Jahan K, Roy SK, Mihrshahi S, Sultana N, Khatoon S, Roy H (2014). Short-term nutrition education reduces low birthweight and improves pregnancy outcomes among urban poor women in Bangladesh. Food Nutr Bull.

[52] Demilew YM, Alene GD, Belachew T (2020). Effect of guided counseling on dietary practices of pregnant women in west Gojjam zone, Ethiopia. PLoS One.

[53] Webb-Girard A, Olude O (2012). Nutrition education and Counselling provided during pregnancy: effects on maternal, neonatal and child health outcomes. Paediatr Perinat Epidemiol.

[54] Zelalem A, Endeshaw M, Ayenew M, Shiferaw S, Yirgu R (2017). Effect of nutrition education on pregnancy specific nutrition knowledge and healthy dietary practice among pregnant women in Addis Ababa. Clin Mother Child Health.

[55] Nikièma L, Huybregts L, Martin-Prevel Y, Donnen P, Lanou HGJ, Offoh P (2017). Effectiveness of facility-based personalized maternal nutrition counseling in improving child growth and morbidity up to 18 months: a cluster-randomized controlled trial in rural Burkina Faso. PLoS One.

[56] Goodarzi-Khoigani M, Baghiani Moghadam MH, Nadjarzadeh A, Mardanian F, Fallahzadeh H, Mazloomy-Mahmoodabad SS (2018). Impact of nutrition education in improving dietary pattern during pregnancy based on pender’s health promotion model: a randomized clinical trial. Iran J Nurs Midwifery Res.

[57] Sun JD, Shao YF, Zhang PL, Li DZ, Gu LY, Guo QN (1990). Evaluation of prenatal nutrition counseling: maternal nutrition status and infant birthweight. Biomed Environ Sci.

[58] Akter SM, Roy SK, Thakur SK, Sultana M, Khatun W, Rahman R (2012). Effects of third trimester counseling on pregnancy weight gain, birthweight, and breastfeeding among urban poor women in Bangladesh. Food Nutr Bull.

