# Supplementary material for: Effects of guided counseling during pregnancy on birth weight of newborns in West Gojjam Zone, Ethiopia: a cluster-randomized controlled trial
Source: BMC Pediatr. 2020 Oct 6;20:466. doi: 10.1186/s12887-020-02363-8 (PMC7542400; doi:10.1186/s12887-020-02363-8)
Supplement: Supplementary file 2 — Additional file 2. [file 12887_2020_2363_MOESM2_ESM.docx]

**Counseling protocol**

**Key messages about diet during pregnancy**

1. Pregnant women need to take a balanced diet composed of all foodstuffs in all food groups every day. Use the following locally available foodstuffs to counsel the women.


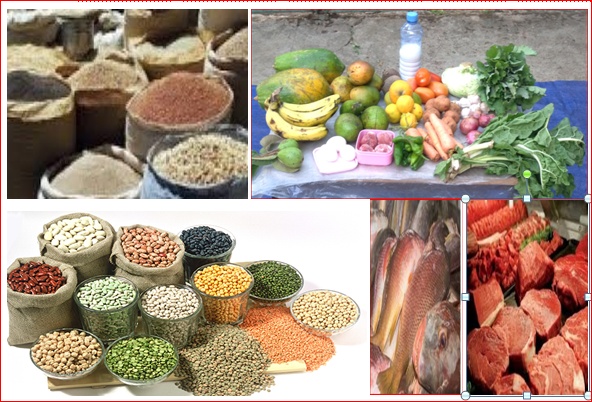


Pregnant women need to eat an adequate amount and balanced diet for the health of themselves and their growing fetus. A balanced diet is a diet which contains carbohydrate, protein, fat, vitamin, and mineral from a variety of foods within and across different food groups. Meaning, it composed of all foodstuffs in all food groups such as cereals, legumes, animal products, vegetables, and fruits.

- Cereals include teff, millet, maize, wheat, barley, sorghum, rye, and rice.
- Legumes include beans, peas, chickpeas, lentils, kidney bean, grass pea, nut, lupine, sunflower seed, linseed, niger seed, and sesame.
- Animal products include meat, eggs, fish, poultry, milk, and milk products.
- Vegetables include carrots, pumpkins, kale, cabbages, tomatoes, Ethiopian collared “gomen”, salad, pepper, onions, and garlic. Roots and tubers also include potatoes, beetroots, and sweet potato.
- Fruits are papaya, bananas, mango, oranges, lemon, avocado, and guava.

Based on their health benefit we can also classify all the above foodstuffs into three classifications as

1. Energy-giving foods: Cereals, fat, and oil are energy-giving foods. Our body needs enough amounts of energy source foods like engines need Kerosene to function properly. The human body needs a continuous regulated supply of nutrients. Energy requirement increase during pregnancy therefore pregnant women needs to increase energy intake with increasing gestational age. Energy is required for all body processes, growth, and physical activity. Thus, unless we take energy source foods we cannot reproduce, move, and live. Even at rest, the body requires energy for muscle contraction, active transport of molecules and ions, and synthesis of macromolecules and other biomolecules from simple precursors.
2. Body-building foods or growing foods: Animal source foods and legumes are good sources of protein. Foods that contain a lot of protein are body-building foods. Protein source foodstuffs are important to build and repair tissues. Protein also used to make enzymes, hormones, and other body chemicals. Protein is an important building block of bones, muscles, cartilage, skin, and blood. Every human being needs protein source foods on a daily base. Pregnant women need an adequate amount and good quality protein for themselves and their growing fetus.
3. Protective foods are foods that contain an adequate amount of vitamins and minerals. Fruits and vegetables are under protective food groups. Like the government trains soldiers to protect the nation’s border, our body produces the immune system to protect our body from outside invaders, such as bacteria, viruses, fungi, and toxins. To develop the immune system your body needs an adequate amount of vitamins, minerals, and proteins. In addition to this, fruits and vegetables are important for physical growth and brain development of the fetus. Therefore, pregnant women should eat an adequate amount and variety of fruits and vegetables.

- After discussing foodstuffs, their classification, and the benefits of taking a variety of foodstuffs with its advantage to improve maternal and fetal health, ask the women about locally available foods and suggest how to prepare and consume a balanced diet using locally available foods.

1. Pregnant women should take at least one additional meal in the second trimester and two additional meals in the third trimester of pregnancy compared with her non-pregnant stat. Discus about the frequency of meals using the experience of a woman who took at least one additional meal in the second trimester and two additional meals in the third trimester of pregnancy.

Pregnant women should take at least one additional meal (4meals) in the second trimester and two additional meals (5 times) in the third trimester of pregnancy compared with her non-pregnant stat. This suggestion is adequate for women whose dietary habit is taking at least three meals before pregnancy. Whereas, when the women took less than three meals before pregnancy it is advisable to take two or more additional meals in the second and three or more meals in the third trimester of pregnancy.

Additionally, the main thing here is her nutritional status, when the woman is undernourished she needs more and also which is not advisable to reduce weight during pregnancy for over nourished women due to fear of the effect of ketone bodies on the fetus. Furthermore, the frequency depends on the amount they took at a time of counseling. Generally, advise the women to take small frequent diet.

The first three months are the period that the fetus's important organs are developing. During this time micronutrient demand for pregnant women is high. To meet this high nutrient requirement, pregnant women should take nutrient-dense foods. All organs developed during the first 12 weeks start increasing size in the next three months or the fetus is growing in length particularly in the second trimester. Thus, pregnant women need to increase energy consumption in addition to their nutrient intakes. During the third trimester, the fetus’s weight increased significantly. This significant weight gain required more energy intake. To meet increased nutrient demand, dietary consumption should be increased with increasing gestational age.

Taking an adequate amount and balanced diet during pregnancy prevents women from malnutrition. It also improves fetal growth and development. It improves gestational weight gain and immune system. It prevents low birth weight, preterm delivery, and postpartum hemorrhage. Therefore, pregnant women should take an adequate amount of a balanced diet for the health of themselves and their growing fetus. When this is the fact your husband and all other relatives such as mother-in-law, and children should co-operate to improve diet during pregnancy.

1. Increase portion size with increasing gestational age. Counsel the women using the following pictures


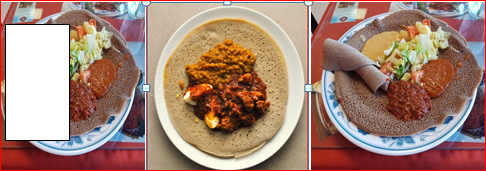


During pregnancy, the nutrient requirement increases with increased gestational age. As a result, pregnant women should increase the portion size of meals per serving with increased gestation. e.g. when women eat half injera or Ethiopian flatbread per serving in the first-trimester of pregnancy, increase it to one injera in the second-trimester and one and a half injera in the third-trimester of pregnancy. This increasing portion size is not only for injera but also for all other foods. Let us take other example roasted cereals. When the women take a half cup of roasted cereals in the first-trimester increase it to one cup in the second and one and a half cup in the third-trimester pregnancy. In general increase the amount of food in each meal through time.

The reason for increasing intake with increasing gestational age is due to increase demand with increasing gestational age because, in the 1st trimester, micro-nutrient supplements are important since the fetus’ functional organs are developing; in the 2nd trimester, the fetus develops in length so mother’s undernutrition in this period is very likely to result in intrauterine stunting; and in the last trimester, the fetus’s weight increases significantly; therefore the mother’s poor weight gain often leads to a low-birth-weight baby. Therefore, increasing portion size enables women to meet increase nutrient and energy demand which in turn improves maternal and fetal wellbeing .

4. Pregnant women should use iodized salt

Iodine is essential for physical growth and brain development in the fetus and young child. So, pregnant women need an adequate amount of iodized salt. Unfortunately, the iodine content of the common foodstuffs in the study area is low. Thus, pregnant women need an additional source of iodine. Iodized salt is a good source of iodine in Ethiopia, hence please advise the woman to use iodized salt during food preparation. Precautions that must be considered during using iodized salt are the following

Buy iodized salt in the shop that is not exposed to sunlight and store it in a closed container (container with a lid) in a dry place away from fire. Add iodized salt after cooking b/c iodine loss is high during cooking.

5. Pregnant women require five serving of fruits and vegetables daily


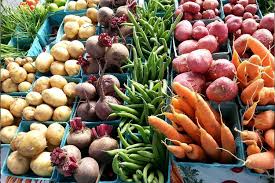

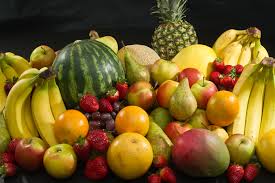

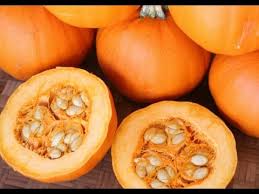

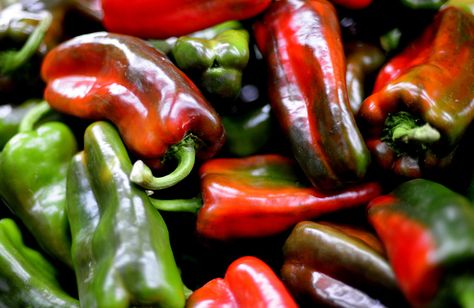

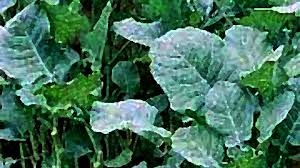

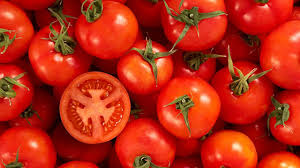


Since nutrient requirements increase during pregnancy, pregnant women need plenty of fruit and vegetables daily. Besides, the immune system weakened during pregnancy. To maintain the immune system, pregnant women need plenty of fruits and vegetables. As the most nutrient-dense of all the food groups, fruit and vegetables provide a comparatively high level of nutrients per calorie. To meet this nutrient demand pregnant women need to take fruits and vegetables five times a day.

Take fruits and vegetables five times a day means include different types of fruits and vegetables on the plate. After counseling about the benefit of taking more fruits and vegetables with its advantage to improve maternal and fetal wellbeing, ask the women about locally available fruits and vegetables and suggest about selection, preparation, and consumption of locally available fruits and vegetables. N.B. counsel women to avoid prolonged cooking of vegetables and to cook vegetables with oil. Moreover, counsel them to choose fresh fruits and vegetables.

6. Pregnant women need to take animal products three times daily


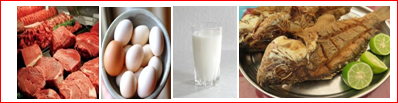


Animal products like meat, fish, milk, and eggs are rich in high-quality protein, vitamins, minerals, and essential fatty acids. These nutrients play a significant role in physical growth, mental growth, and development of the fetus. The requirement of these nutrients increased during pregnancy, to meet increased demand, pregnant women need animal products three times a day.

Comparison of the benefits of taking adequate amount of balanced diet and the consequences of taking inadequate amount or unbalanced diet during pregnancy.

Taking an adequate amount and a balanced diet improve gestational weight gain and birth weight. It prevents maternal undernutrition, intrauterine growth retardation, preterm birth, postpartum hemorrhage, infection, neonatal and maternal mortality. Whereas, taking an inadequate amount and/ or an unbalanced diet leads to having maternal undernutrition, intrauterine growth retardation, low birth weight baby and preterm birth, postpartum hemorrhage, infection, neonatal and maternal mortality.

7. Pregnant women should take iron/folic acid supplement

Since pregnant women don’t get an adequate amount of iron from their diet, they need iron and folic acid supplement to meet their own nutritional needs as well as those of the developing fetus. Iron/folic acid supplement prevents women from anemia and it has a significant effect on physical growth and brain development of the growing fetus. A pregnant woman who has no anemia needs one tablet with 30 mg to 60 mg of elemental iron and 400 µg (0.4 mg) folic acid daily oral iron and folic acid supplementation. Start Iron supplement as early as possible and given throughout pregnancy or take at least for 3-6months. Taking iron/folic acid supplement prevent maternal anemia, puerperal sepsis, low birth weight, and preterm birth. Whereas, anemic women need treatment dose of iron/folic acid based on the physician’s order. Counsel the women using the following picture.

8. Pregnant women should use an impregnated bed net

An immune system weakened during pregnancy. This makes pregnant women at risk of acquired infection. To prevent malaria pregnant women should use an impregnated mosquito net. They need to use insecticide-treated nets because malaria in pregnancy leads to low birth weight, premature birth, anemia, maternal and neonatal mortality. The use of insecticide-treated nets (ITNs) during pregnancy is one of the proven interventions to reduce the malaria burden. Moreover, she should keep her personal and environmental hygiene.

9. Pregnant women should reduce heavy workload and take rest during the day

Since heavy workload increases energy demands which farther increased the nutrient demand of pregnant women, they should reduce heavy workload during pregnancy. Furthermore, to reduce nutrient demand during pregnancy, pregnant women need to take 1-2hours day time rest and 8hours rest in the night.

10. Attained ANC service

Antenatal care is a type of preventive healthcare. Its goal is to provide regular check-ups of woman’s health to treat and prevent potential health problems throughout pregnancy and to promote healthy lifestyles that benefit both mother and child. So, the woman should attend four or more antenatal care services.

10. Counsel the woman by comparing the experience of two women. One woman took an adequate amount and diversified meals whereas the other woman took an inadequate amount and a monotonous diet. The woman who took adequate amount and diversified meals was healthy and her infant was also healthy whereas a woman who consumed an inadequate amount and undiversified meals had a history of stillbirth.

During counseling ask the women about her income and barriers that interfere here from taking adequate and diversified meals. After identifying gaps inhibit her from taking an adequate meal, discuss the solutions to solve the identified barriers. Identify family members who involve in encouraging women to take adequate amounts and diversified meals.

These key messages are prepared by a Bahir Dar University PhD student Yeshalem Mulugeta to improve maternal diet during pregnancy.
