# Supplementary figures and images for: Effects of guided counseling during pregnancy on birth weight of newborns in West Gojjam Zone, Ethiopia: a cluster-randomized controlled trial
Source: BMC Pediatr. 2020 Oct 6;20:466. doi: 10.1186/s12887-020-02363-8 (PMC7542400; doi:10.1186/s12887-020-02363-8)

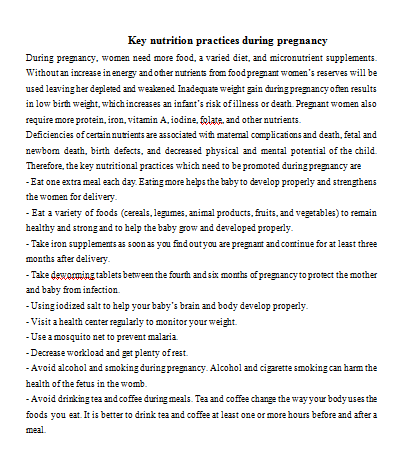

Supplement: Supplementary file 3 — Additional file 3. [file 12887_2020_2363_MOESM3_ESM.docx]
